# The association between systemic immune-inflammation index and mortality of elderly chronic bronchitis patients complicated by acute myocardial infarction

**DOI:** 10.3389/fcvm.2026.1696646

**Published:** 2026-05-25

**Authors:** Chao Zhang, Yunfeng Ni, Dong Han

**Affiliations:** 1 The Sixth Department of Health Care, The Second Medical Center & National Clinical Research Center for Geriatric Diseases, Chinese PLA General Hospital, Beijing, China; 2 Department of Thoracic Surgery, Tangdu Hospital, The Airforce Medical University, Xi ‘an City, Shanxi, China

**Keywords:** acute myocardial infarction, chronic bronchitis, elderly patients, mortality, systemic immune-inflammation index

## Abstract

**Objectives:**

To explore the relationship between the systemic immune-inflammation index (SII) and in-hospital mortality in elderly individuals diagnosed with chronic bronchitis (CB) complicated by acute myocardial infarction (AMI).

**Methods:**

This retrospective observational study included 252 elderly patients diagnosed with CB complicated by AMI, who were consecutively enrolled based on standardized diagnostic criteria. Patients were categorized into a survival group (*n* = 204) and a mortality group (*n* = 48) according to in-hospital outcomes. Demographic, clinical, and laboratory parameters collected at admission were extracted from electronic medical records for analysis, and the SII was calculated for all subjects. Group differences were analyzed using the Mann–Whitney *U* test, *t*-test, or chi-square test, selected in accordance with the distributional characteristics of the data. Parameters exhibiting significant intergroup variation underwent univariate logistic regression analysis, and variables with notable associations were subsequently introduced into a multivariate regression model. Finally, ROC curve analysis was conducted to determine the prognostic value of the SII in predicting the risk of in-hospital mortality.

**Results:**

Overall patient characteristics showed that non-survivors had markedly higher inflammatory markers and altered lipid profiles compared with survivors. Analysis using univariate logistic regression revealed strong associations of Hb, TC, LDL-C, CRP, and SII with the likelihood of mortality. Multivariate logistic regression identified LDL-C (OR = 29.174, *P* < 0.001), CRP (OR = 1.135, *P* < 0.001), and SII (OR = 1.001, *P* < 0.001) as independent predictors of in-hospital mortality. ROC analysis demonstrated that SII had strong discriminative ability (AUC = 0.864), and the combination of SII, CRP, and LDL-C yielded the highest predictive performance (AUC = 0.935).

**Conclusion:**

Among elderly individuals suffering from CB in combination with AMI, a higher SII emerged as an independent determinant of in-hospital mortality.

## 1 Introduction

Chronic bronchitis (CB), a major phenotype of chronic obstructive pulmonary disease (COPD), is a common and disabling respiratory disorder among the elderly population (1). In routine clinical settings, CB is typically characterized by a prolonged cough with expectoration that persists for a total period of no fewer than three months within a single year and recurs in successive years for at least two years. This condition is often accompanied by repeated infections of the respiratory tract, gradual decline in airflow, and evidence of systemic inflammatory responses (2). In elderly patients with CB, the natural decline of lung function together with reduced immune capacity makes them particularly vulnerable to acute disease exacerbations as well as additional comorbid illnesses. These combined factors contribute to a markedly higher likelihood of hospital admission and an elevated risk of death (3, 4).

Within older adults, AMI continues to constitute a critical cardiovascular condition that poses substantial threats to health, ranking among the foremost contributors to both death and persistent disability on a global scale (5, 6). The incidence of AMI rises sharply with advancing age, and elderly patients often present with atypical symptoms, delayed diagnosis, and a higher prevalence of comorbidities, which contribute to poor clinical outcomes (5, 6). Recent studies have highlighted a close and complex interaction between chronic respiratory diseases and cardiovascular disorders. Notably, individuals diagnosed with CB or COPD are reported to carry a higher likelihood of experiencing AMI. This association is thought to be linked with persistent systemic inflammation, oxidative stress, impairment of endothelial function, and heightened platelet reactivity (7, 8). When CB occurs together with AMI, the overlap of persistent and acute inflammatory responses may aggravate cardiovascular damage, disturb normal immune homeostasis, and contribute to a higher probability of in-hospital death (9).

An enhanced understanding of systemic inflammation highlights its role as a central pathophysiological process that bridges chronic airway pathology and the development of atherosclerotic cardiovascular disease (10). In routine clinical practice, traditional inflammatory indices, including CRP and WBC counts, are commonly applied to evaluate the degree of systemic inflammation and to provide guidance in estimating cardiovascular risk (11, 12). Nevertheless, conventional markers often fail to provide a full representation of the complex interactions that occur among innate immunity, adaptive immune responses, and thrombotic processes. In contrast, the SII, a recently introduced composite parameter derived from the formula platelet count × neutrophil count ÷ lymphocyte count, combines three peripheral blood cell measures and offers an integrated perspective on inflammatory status, immune regulation, and coagulation-related activity (13). The SII has been reported to hold prognostic value across a range of diseases, including malignant tumors (14, 15), septic conditions (16), and several cardiovascular disorders such as heart failure (17) and acute coronary syndromes (18). Elevated SII levels are generally linked with greater disease burden, unfavorable clinical trajectories, and a higher likelihood of mortality (17–19). Furthermore, recent evidence highlights that higher SII is independently associated with an increased risk of mortality and rehospitalization in patients with heart failure with preserved ejection fraction (HFpEF), providing a relevant pathophysiological bridge to chronic bronchitis given their shared features of sustained systemic inflammation and multimorbidity (20).

Although growing evidence indicates that the SII carries meaningful prognostic implications in cardiovascular pathology, its clinical significance in elderly individuals suffering from CB complicated by AMI has not yet been fully clarified. We specifically targeted this cohort to investigate a unique “double-hit” inflammatory pathophysiology. In this highly vulnerable demographic, the baseline chronic, low-grade systemic inflammation inherent to CB is acutely compounded by the severe thrombo-inflammatory cascade of an AMI, while age-related immunosenescence further contributes to a specific immune imbalance. Considering the unique pathophysiological mechanisms and the generally reduced physiological resilience of this patient group, the ability to identify, at an early stage, those at elevated risk of in-hospital death is of considerable relevance for clinical management. Accordingly, the present investigation aimed to explore the relationship between SII and mortality in elderly patients with CB and AMI, while also assessing its prognostic performance relative to traditional inflammatory biomarkers and standard biochemical indicators.

## 2 Methods

### 2.1 Subjects

This investigation adopted a retrospective design and enrolled 252 elderly individuals diagnosed with CB in combination with AMI, who were consecutively hospitalized in the Departments of Cardiology and Respiratory Medicine at The Second Medical Center & National Clinical Research Center for Geriatric Diseases, Chinese PLA General Hospital from February 2020 to May, 2025. CB was diagnosed in line with the WHO criteria, which require cough with sputum production lasting for no less than three months per year across two successive years. AMI was identified according to the currently endorsed international definition, based on an integrated evaluation of typical clinical presentation, electrocardiographic abnormalities, and elevated cardiac biomarkers. On the basis of in-hospital outcomes, cases were categorized into a survival cohort (*n* = 204) and a mortality cohort (*n* = 48). The primary endpoint of this investigation was all-cause in-hospital mortality. Among the 48 non-survivors, the clinical progression to death was predominantly characterized by refractory cardiogenic shock and progressive multi-organ dysfunction, pathways reflecting a severe systemic thrombo-inflammatory burden. Specifically, 16 patients (33.3%) in the mortality group experienced documented ventricular tachycardia or ventricular fibrillation (VT/VF) as part of their terminal clinical course. Notably, no patients were excluded or reclassified based on the specific terminal mechanism of death. Data regarding demographic characteristics, predominant symptoms, coexisting diseases, and laboratory parameters at admission were systematically retrieved from the electronic medical record system. Ethical approval was approved by The Second Medical Center & National Clinical Research Center for Geriatric Diseases, Chinese PLA General Hospital, and informed consent was signed by all subjects.

### 2.2 Inclusion and exclusion criteria

To ensure cohort representativeness and minimize selection bias, patients were retrospectively and consecutively enrolled by querying the hospital's electronic medical record (EMR) system. Specifically, we first identified consecutive elderly patients admitted for AMI, and subsequently cross-referenced their prior medical records for a pre-existing history of CB. The eligibility criteria for patient enrollment were defined as follows: (1) age of 65 years or older; (2) a previously established and definite diagnosis of CB based on the WHO standards documented prior to the index AMI event, requiring a clinical history of cough with sputum persisting for no less than three months per year over a period of two consecutive years; (3) meeting the diagnostic definition of AMI at the time of current admission, which entailed the presence of characteristic clinical symptoms in combination with electrocardiographic abnormalities and elevated cardiac biomarkers, in accordance with the widely accepted international guidelines. Crucially, AMI must have been the primary indication for the current hospital admission, rather than a secondary complication arising during a hospitalization initially intended for an acute exacerbation of CB or other acute conditions. Furthermore, only those patients with complete demographic records, clinical characteristics, and laboratory data documented at the time of hospital admission were included in the analysis.

Patients were excluded as: (1) evidence of acute or chronic infections other than CB upon admission; (2) the presence of autoimmune disorders, hematological diseases, or systemic inflammatory conditions; (3) advanced impairment of hepatic or renal function; (4) active malignant disease or a cancer history within the preceding five years; (5) recent administration of corticosteroids or other immunosuppressive drugs; and (6) incomplete medical documentation or missing laboratory parameters required for SII calculation.

### 2.3 Clinical data collection

Clinical information for all enrolled patients was retrospectively derived from the electronic medical record system. The demographic profile encompassed variables such as age, sex, and BMI. Primary symptoms recorded at admission, including cough, dyspnea, chest tightness, wheezing, and purulent sputum, were thoroughly documented. Lifestyle characteristics, with special attention to smoking history, along with coexisting disorders, e.g., such as diabetes mellitus, cerebrovascular disorders, hypertension, osteoporosis, and malignancies, were incorporated into the dataset. Within 24 h of admission, peripheral venous blood samples were collected for laboratory evaluation. Hematological measurements consisted of Hb, platelet counts, WBC counts, neutrophil counts, and lymphocyte counts. Biochemical analyses included BUN, SCr, Alb, TG, TC, LDL-C, and HDL-C. Markers of CRP and the SII, the latter determined by the formula: platelet count × neutrophil count ÷ lymphocyte count.

### 2.4 Statistical analysis

Statistical analyses were carried out using SPSS software, version 26.0 (IBM Corp., Armonk, NY, USA). The distribution patterns of continuous variables were initially examined with the Kolmogorov–Smirnov test to evaluate normality. When data conformed to a normal distribution, mean ± SD was used, and independent-samples *t*-test was performed for intergroup comparisons. For data not normally distributed, results were expressed as median with IQR, and intergroup differences were assessed through the Mann–Whitney *U* test. Categorical variables were analyzed by either Fisher's exact test or *χ*^2^ test, depending on suitability. Subsequently, significant variables revealed by univariate analysis were included in logistic regression. To enhance the clinical interpretability of the odds ratios (ORs), the continuous variable SII was rescaled to reflect a per 500-unit increase. Furthermore, to strictly adhere to the “10 events per variable (EPV)” rule and prevent model overfitting given the limited number of outcome events (*n* = 48), a parsimonious, fully prespecified multivariate logistic regression model was constructed. Instead of introducing all significant univariate variables, the final multivariate model prioritized and included only three key predictors: rescaled SII, CRP, and LDL-C. ORs together with 95% CIs were calculated for these specific variables.

Furthermore, ROC curve analysis was performed to assess the predictive accuracy of the SII for in-hospital mortality. The AUC with its 95% CI, along with the optimal cutoff value indicated by the Youden index, were reported, as well as the associated sensitivity and specificity. All tests were two-tailed, and *P* < 0.05 indicated significant difference. Multicollinearity among independent variables was evaluated using variance inflation factors (VIF), and no variable exceeded the commonly accepted threshold, indicating an absence of significant collinearity. For the construction of the combined predictive model, the predicted probabilities generated by the logistic regression model incorporating rescaled SII, CRP, and LDL-C were used as the composite indicator. The goodness-of-fit of the updated parsimonious logistic regression model was assessed using the Hosmer–Lemeshow test, indicating excellent calibration of the combined model (*χ*^2^ = 2.375, *P* = 0.967).

## 3 Results

### 3.1 Baseline information of survivor and mortality groups in elderly CB patients complicated with AMI

In total, 252 elderly patients diagnosed with CB complicated by AMI were retrospectively analyzed, including 204 cases in the survival group and 48 in the mortality group (Table 1). Baseline characteristics, such as age, sex, BMI, smoking history, comorbid conditions, and primary symptoms at admission, were comparable between the two cohorts, with no significant differences (*P* > 0.05). Compared with survivors, patients in the mortality group demonstrated a pronounced reduction in lymphocyte count (0.94 ± 0.35 vs. 1.43 ± 0.32 × 10^9^/L, *P* < 0.001). By contrast, platelet levels (265.33 ± 39.53 vs. 250.29 ± 42.19 × 10^9^/L, *P* = 0.025) and hemoglobin concentrations (138.15 ± 9.25 vs. 133.91 ± 10.34 g/L, *P* = 0.010) were significantly elevated. With respect to lipid metabolism, non-survivors exhibited higher TC (4.92 ± 0.59 vs. 4.55 ± 0.58 mmol/L, *P* < 0.001) and LDL-C (3.11 ± 0.43 vs. 2.54 ± 0.41 mmol/L, *P* < 0.001) compared with survivors, whereas HDL-C and TG showed no meaningful group differences (*P* > 0.05). Inflammatory parameters also diverged: both CRP (25.13 ± 9.90 vs. 18.23 ± 5.85 mg/L, *P* < 0.001) and the SII (2,854.73 ± 1,457.19 vs. 1,575.96 ± 648.89 × 10^9^/L, *P* < 0.001) were markedly higher in the mortality group. Conversely, no significant variation was detected between groups for WBC, neutrophil levels, SCr, Alb, or BUN (*P* > 0.05 for all).

**Table 1:** Comparison of baseline characteristics between survivor and mortality groups in elderly CB patients complicated by AMI.

Indices	Survivor group (*n* = 204)	Mortality group (*n* = 48)	*P* value
Age (years)	73.11 ± 4.98	74.10 ± 6.21	0.239
Gender [*n* (%)]			0.708
Female	49 (24.02)	10 (20.83)	
Male	155 (75.98)	38 (79.17)	
BMI (kg/m^2^)	24.97 ± 3.90	24.52 ± 4.78	0.484
Smoking [*n* (%)]	159 (77.94)	39 (81.25)	0.699
Comorbidities [*n* (%)]			
Diabetes	161 (78.92)	40 (83.33)	0.556
Cerebrovascular disease	35 (17.16)	7 (14.58)	0.83
Osteoporosis	16 (7.84)	5 (10.42)	0.773
Cured malignancies	16 (7.84)	3 (6.25)	0.777
HTN	120 (58.82)	31 (64.48)	0.515
Symptoms [*n* (%)]			
Cough	182 (89.22)	44 (91.67)	0.794
Dyspnea	126 (61.76)	29 (60.42)	0.87
Purulent sputum	37 (18.14)	10 (20.83)	0.401
Chest distress	39 (19.12)	10 (20.83)	0.84
Wheezing	45 (22.06)	12 (25.00)	0.702
Laboratory testing			
WBC (×10^9^/L)	10.43 ± 1.56	10.28 ± 1.43	0.549
Neutrophils (×10^9^/L)	8.41 ± 1.17	8.64 ± 1.07	0.211
Lymphocytes (×10^9^/L)	1.43 ± 0.32	0.94 ± 0.35	<0.001
Plt (×10^9^/L)	250.29 ± 42.19	265.33 ± 39.53	0.025
Hb (g/L)	133.91 ± 10.34	138.15 ± 9.25	0.01
TC (mmol/L)	4.55 ± 0.58	4.92 ± 0.59	<0.001
HDL (mmol/L)	1.14 ± 0.20	1.12 ± 0.19	0.596
LDL (mmol/L)	2.54 ± 0.41	3.11 ± 0.43	<0.001
TG (mmol/L)	4.68 ± 0.67	4.72 ± 0.62	0.707
SCr (umol/L)	75.40 ± 10.05	74.18 ± 11.87	0.467
Alb (g/L)	34.36 ± 4.02	33.68 ± 4.75	0.305
BUN (mmol/L)	5.66 ± 0.90	5.83 ± 1.07	0.261
CRP (mg/L)	18.23 ± 5.85	25.13 ± 9.90	<0.001
SII (×10^9^/L)	1,575.96 ± 648.89	2,854.73 ± 1,457.19	<0.001

CB, chronic bronchitis; AMI, acute myocardial infarction; BMI, body mass index; HTN, hypertension; WBC, white blood cell; Plt, platelet; Hb, hemoglobin; TC, total cholesterol; HDL, high-density lipoprotein; LDL, low-density lipoprotein; TG, triglyceride; SCr, serum creatinine; Alb, albumin; BUN, blood urea nitrogen; CRP, C-reactive protein; SII, systemic immune-inflammation index.

### 3.2 Logistic regression analysis of risk factors for mortality in elderly CB patients complicated by AMI

The univariate logistic regression analysis indicated significant associations between in-hospital mortality and Hb, TC, LDL, CRP, as well as the SII (Table 2). Elevated levels of Hb (95% CI: 1.010–1.077, OR = 1.043, *P* = 0.011), TC (95% CI: 1.709–5.585, OR = 3.090, *P* < 0.001), LDL (OR = 29.155, 95% CI: 10.587–80.284, *P* < 0.001), CRP (95% CI: 1.084–1.190, OR = 1.136,*P* < 0.001), and SII (95% CI: 1.001–1.002, OR = 1.001,*P* < 0.001) each suggested an increased likelihood of in-hospital death. To adhere to the “10 events per variable (EPV)” rule and mitigate the risk of model overfitting due to the limited number of mortality events, a parsimonious multivariate regression model was adopted. Consequently, Hb and TC were excluded from further analysis. The fully prespecified multivariate model prioritized three key predictors: LDL, CRP, and a rescaled SII (analyzed per 500-unit increase to enhance clinical interpretability). Within this optimized framework, LDL (95% CI: 9.915–122.953, OR = 31.681, *P* < 0.001), CRP (95% CI: 1.065–1.219, OR = 1.135, *P* < 0.001), and the rescaled SII (95% CI: 1.557–3.025, OR = 2.109, *P* < 0.001) continued to demonstrate independent prognostic significance (Table 3).

**Table 2:** Univariate logistic regression analyses identifying risk factors associated with mortality in elderly CB patients complicated by AMI.

Variables	*B*	SE	Wald *X*^2^	P	OR	95% CI
Hb	0.042	0.016	6.49	0.011	1.043	1.010–1.077
TC	1.128	0.302	13.946	<0.001	3.09	1.709–5.585
LDL	3.373	0.517	42.584	<0.001	29.155	10.587–80.284
CRP	0.127	0.024	28.272	<0.001	1.136	1.084–1.190
SII	0.001	<0.001	35.664	<0.001	1.001	1.001–1.002

CB, chronic bronchitis; AMI, acute myocardial infarction; Hb, hemoglobin; TC, total cholesterol; LDL, low-density lipoprotein; CRP, C-reactive protein; SII, systemic immune-inflammation index.

**Table 3:** Multivariate logistic regression analyses identifying independent risk factors associated with mortality in elderly CB patients complicated by AMI.

Variables	*B*	SE	Wald *X*^2^	*P*	OR	95% CI
LDL	0.746	0.169	19.419	<0.001	31.681	9.915–122.953
CRP	0.126	0.034	13.532	<0.001	1.135	1.065–1.219
SII	3.456	0.637	29.429	<0.001	2.109	1.557–3.025

CB, chronic bronchitis; AMI, acute myocardial infarction; Hb, hemoglobin; TC, total cholesterol; LDL, low-density lipoprotein; CRP, C-reactive protein; SII, systemic immune-inflammation index.

### 3.3 ROC analysis for predicting mortality in elderly CB patients complicated by AMI

**Figure 1:**
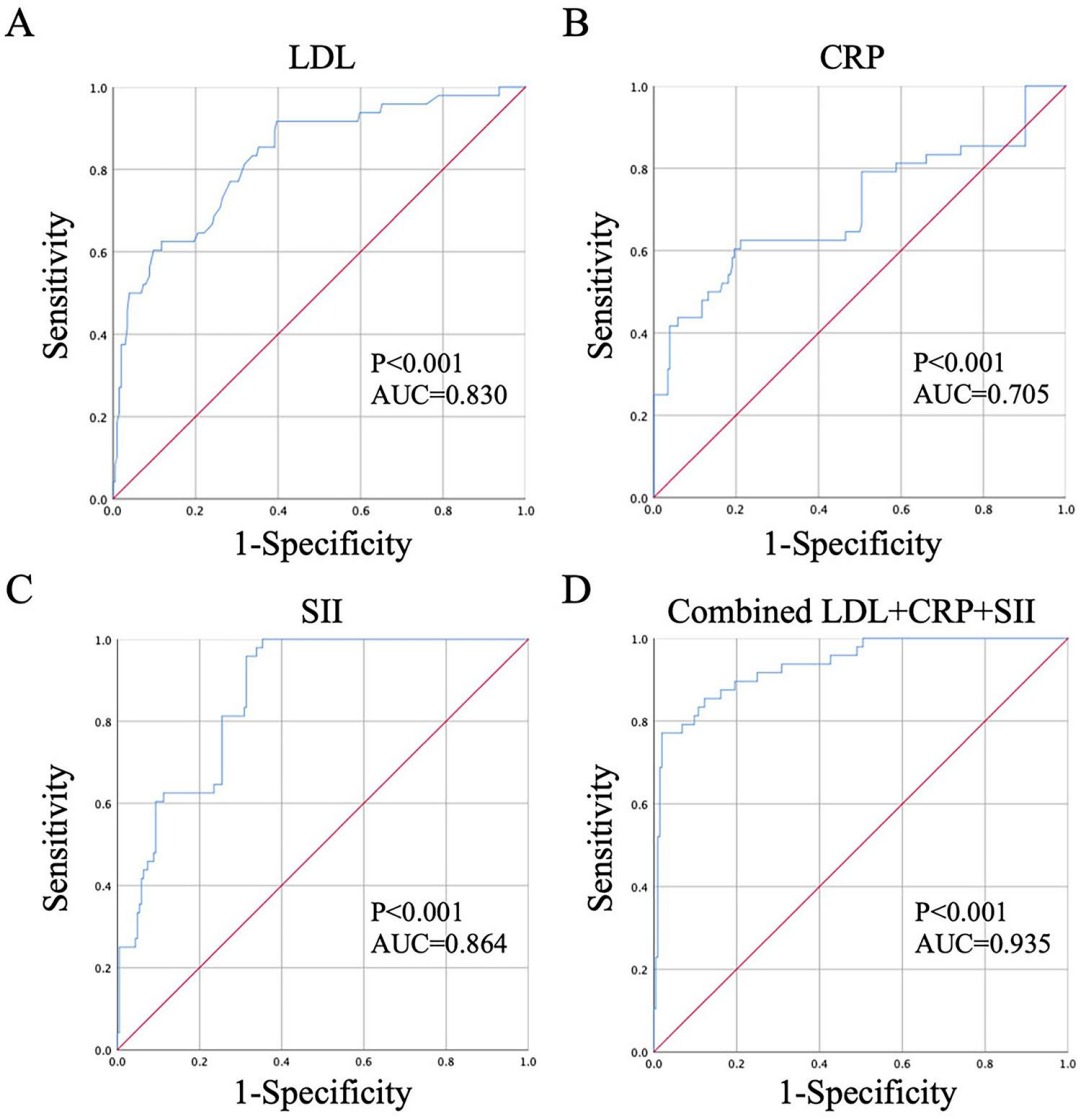
ROC analysis of **(A)** LDL, **(B)** CRP, **(C)** SII and **(D)** their combination for predicting mortality in elderly CB patients complicated by AMI. LDL, low-density lipoprotein; CRP, C-reactive protein; SII, systemic immune-inflammation index; AUC, area under the curve; ROC, receiver operating characteristic; CB, chronic bronchitis; MI, myocardial infarction.

Table 4 and Figure 1 illustrate that LDL achieved an AUC of 0.830 (95% CI: 0.764–0.897, *P* < 0.001) for predicting in-hospital mortality, with an optimal threshold of 2.645 mmol/L, corresponding to a sensitivity of 91.7% and a specificity of 60.3%. CRP demonstrated only moderate discriminative capacity (AUC = 0.705, 95% CI: 0.609–0.801, *P* < 0.001; cutoff = 21.45 mg/L; sensitivity 62.5%, specificity 78.9%). By contrast, the SII provided superior predictive performance (AUC = 0.864, 95% CI: 0.818–0.911, *P* < 0.001; cutoff = 1,655.58 × 10^9^/L; sensitivity 95.8%, specificity 68.6%). When these three markers were integrated into a composite model, the discriminative accuracy reached its highest value (AUC = 0.935, 95% CI: 0.897–0.974, *P* < 0.001), with sensitivity of 77.1% and specificity as high as 98.0%. Overall, these results suggest that LDL, CRP, and SII individually confer prognostic significance, while their combined use yields even greater predictive precision.

**Table 4:** ROC analysis of SII for predicting mortality in elderly CB patients complicated by AMI.

Variables	AUC	95% CI	Best cut-off value	Sensitivity (%)	Specificity (%)	*P* value
LDL	0.83	0.764–0.897	2.645	91.7	60.3	<0.001
CRP	0.705	0.609–0.801	21.45	62.5	78.9	<0.001
SII	0.864	0.818–0.911	1,655.58	95.8	68.6	<0.001
Combined	0.935	0.897–0.974		77.1	98	<0.001

CB, chronic bronchitis; AMI, acute myocardial infarction; LDL, low-density lipoprotein; CRP, C-reactive protein; SII, systemic immune-inflammation index; AUC, area under the curve; CI, confidence interval.

## 4 Discussion

This retrospective study of 252 elderly patients with CB and AMI examined the association between SII and in-hospital mortality. Baseline demographics, comorbidities, and admission symptoms were similar between survivors and non-survivors, whereas several laboratory parameters—including lymphocyte and platelet counts, Hb, lipid indices, CRP, and SII—showed significant differences. In univariate analysis, Hb, TC, LDL-C, CRP, and SII were associated with mortality, but only LDL-C, CRP, and SII remained independent predictors after multivariable adjustment. ROC analysis further showed that SII had stronger discriminative ability than CRP or LDL-C alone, and the combined model incorporating all three markers yielded the highest predictive accuracy.

The coexistence of CB and AMI in elderly patients reflects a complex interplay between chronic airway inflammation, systemic immune dysregulation, and cardiovascular injury (9, 21–23). Chronic bronchitis, characterized by persistent airway inflammation, mucus hypersecretion, and recurrent infections (23, 24), can induce sustained systemic inflammation and oxidative stress, accompanied by elevated cytokines such as IL-6 and TNF-α (25–27). These inflammatory mediators contribute to endothelial dysfunction, reduced nitric oxide bioavailability, and vascular remodeling, thereby accelerating atherosclerosis (28, 29). Chronic hypoxemia and hypercapnia in CB further exacerbate oxidative stress and myocardial oxygen imbalance, increasing susceptibility to ischemic events (30–32). Conversely, AMI induces a systemic inflammatory cascade characterized by neutrophil activation, cytokine release, and increased platelet aggregation, thereby worsening myocardial injury and ventricular dysfunction (33–35). In patients with coexisting CB and AMI, chronic airway inflammation further primes endothelial and immune responses, amplifying thrombo-inflammatory activity during acute ischemia (22, 36). This synergistic “double-hit” effect may explain the heightened inflammatory markers and poorer outcomes in non-survivors. Reduced lymphocyte counts and elevated platelet levels reflect immune suppression and intensified thrombo-inflammation, consistent with their known associations with adverse cardiovascular prognosis and coronary thrombosis (37–39). Higher hemoglobin levels in non-survivors may indicate hypoxemia-related hemoconcentration, which can increase blood viscosity and exacerbate myocardial ischemia in elderly patients (40, 41). Collectively, these mechanisms illustrate how chronic bronchial inflammation and acute myocardial injury interact to elevate mortality risk.

As an emerging biomarker, SII simultaneously reflects inflammatory activity, immune competence, and thrombotic potential within a single index. In contrast to traditional measures, such as the NLR or PLR, SII is a more integrated and comprehensive choice for the evaluation of immune–inflammatory balance. Evidence from previous research has highlighted its prognostic relevance in oncology, where higher SII values have been linked with tumor progression, limited therapeutic response, and poorer survival outcomes across several cancer types, including lung (42, 43), gastrointestinal (44, 45), and hepatocellular malignancies (14, 46). In addition, SII has been investigated in infectious and critical care settings, with evidence suggesting its predictive utility in sepsis (47), severe pneumonia (48), and intensive care mortality (49). In cardiovascular disease, growing evidence indicates that higher SII levels correlate with adverse outcomes in CAD, ACS, and HF, predicting increased MACE risk, poorer prognosis after PCI, and higher short-term mortality in AMI patients (50, 51). The present study further suggests that SII may capture the interplay among inflammation, immune imbalance, and pro-thrombotic processes in elderly patients with CB complicated by AMI—a population particularly susceptible due to overlapping chronic airway inflammation and cardiovascular vulnerability. Our findings show that elevated SII is independently associated with in-hospital mortality, and ROC analysis indicates that SII outperforms CRP alone, while the combined model with CRP and LDL-C provides optimal predictive accuracy. Overall, these results support SII as a practical, low-cost, and accessible biomarker that may enhance risk stratification in this high-risk group.

Alongside SII, both CRP and LDL-C were identified as independent predictors of in-hospital mortality. CRP, an established acute-phase reactant produced by hepatocytes in response to IL-6 and other pro-inflammatory mediators (52), not only reflects systemic inflammation but may also contribute directly to vascular injury by promoting endothelial dysfunction, oxidative stress, and monocyte infiltration into atherosclerotic plaques (53, 54). In CB, chronic airway inflammation may sustain elevated CRP levels, which, when compounded by the acute inflammatory surge of AMI, can intensify systemic inflammatory responses and worsen prognosis. Elevated CRP has consistently been associated with larger infarct size, adverse ventricular remodeling, and higher mortality in AMI patients (55, 56), findings echoed by the higher CRP levels observed in non-survivors in our cohort. LDL-C likewise remains a central marker of cardiovascular risk. Elevated LDL-C facilitates atherosclerosis through lipid deposition, oxidative modification, and foam cell formation (57, 58) and contributes to plaque vulnerability and rupture—the precipitating event in AMI (59). In elderly CB patients, systemic inflammation and oxidative stress may further accelerate LDL-C oxidation, enhancing its pathogenic role. Notably, LDL-C showed the strongest association with mortality in our study, with an odds ratio exceeding 29, underscoring the importance of lipid metabolism in shaping outcomes and suggesting a potential role for more aggressive lipid-lowering strategies in this high-risk population.

### Limitations

This investigation has certain limitations. First, its retrospective, single-center design may introduce selection bias and restrict external applicability. Second, we only assessed in-hospital mortality; evaluating long-term outcomes (e.g., recurrent events) requires future extended follow-ups. Third, retrospective constraints precluded full adjustment for key unmeasured confounders (LVEF, Killip class, precise PCI timing, FEV₁/FVC, nutrition, and medications), which may influence baseline SII and outcomes. Additionally, AMI patients were not stratified into NSTEMI and STEMI, warranting future subgroup analyses. Fourth, CB diagnosis relied on symptom-based criteria without spirometry (contraindicated during acute AMI), meaning diagnostic overlap with conditions like COPD or asthma—and their inherent systemic inflammation—cannot be fully excluded. Fifth, despite utilizing a parsimonious model, the unusually high odds ratio and wide confidence interval for LDL-C likely resulted from the small sample size and limited mortality events (*n* = 48), necessitating cautious interpretation and validation in larger cohorts. Finally, our results may not generalize to patients with complex coronary anatomies, as all subjects strictly underwent primary PCI. Moreover, given the exploratory nature of this study, interpreting the clinical relevance of incremental SII changes requires further large-scale, longitudinal investigations with dynamic monitoring.

## 6 Conclusion

In summary, the SII emerged as an independent indicator of in-hospital mortality among elderly patients with CB complicated by AMI. When considered alongside CRP and LDL-C, SII enhanced predictive accuracy, reflecting the interconnection between systemic inflammation, immune imbalance, and lipid abnormalities in this vulnerable group. These observations suggest that incorporating SII into clinical evaluation frameworks may facilitate earlier risk stratification and help inform more tailored management approaches.

## Data Availability

The original contributions presented in the study are included in the article/Supplementary Material, further inquiries can be directed to the corresponding author.
